# Validity of information integration based on subjective and physiological data from a real sports condition: application to the judgment of fatigue in sport

**DOI:** 10.3389/fspor.2024.1338883

**Published:** 2024-03-04

**Authors:** Alban Legall, Anne-Fleur Gaston, Eric Fruchart

**Affiliations:** Laboratoire Interdisciplinaire Performance Santé Environnement de Montagne (LIPSEM) - UR 4604, University of Perpignan Via Domitia, Font-Romeu, France

**Keywords:** validity, information integration, fatigue, training impulse, interdisciplinary

## Abstract

The objective of the present study was to confirm the convergent validity of information integration theory in the judgment of fatigue in sport, using information integration, subjective, and physiological data. Twenty healthy athletes were confronted with six cycling scenarios in two experimental conditions. In the laboratory condition, the athletes imagined the scenarios and had to cognitively combine the exercise intensity (30%, 50%, and 70% of the maximal intensity) and the exercise duration (15 and 30 min) when judging their expected level of fatigue. In the real sports condition, the athletes enacted each scenario and then rated their subjective fatigue. The heart rate was recorded continuously, so that the physiological training impulse could be calculated. We applied analyses of variance to the data and analyzed correlations between variables. The information integration data from the laboratory condition, the subjective data from the real sports condition, and the objective (physiological) data from the real sports condition were strongly correlated. The information integration patterns concerning fatigue as a function of the exercise duration and intensity obtained respectively from the three data sets were extremely similar.

## Introduction

Over the last few decades, sports trainers have started to consider the athlete as a whole. A variety of physiological and psychological indicators are now used to monitor an individual's level of fatigue and thus optimize his/her physical performance (1). Fatigue occurs when the energy produced by the body is no longer sufficient to respond to an external stimulus, such as physical activity (2). Fatigue leads to a decrease in physical performance (3), and extreme fatigue may lead to injury (4). Hence, fatigue monitoring is a central issue for sports coaches (5).

Training imposes stress on athletes and shifts their physical and psychological well-being along a continuum from acute fatigue to overreaching (6). While overreaching may be incorporated with care into a periodized training plan, progression towards overtraining syndrome is undesirable. Appropriate titration of fatigue is important for both adaptation to training and performance in competition (7). Athletes should be monitored closely to ensure that training elicits the desired effects on their well-being and performance. In order to estimate the level of fatigue induced by training, coaches can rely on objective (physiological) data and/or subjective (psychological) data (8). Coaches can calculate the training load by using various methods based on physiological data (9). For example, the training impulse (TRIMP) is often considered to be a useful means of objectively assessing the training load and the level of fatigue (7). A TRIMP is a unit of physical effort that is calculated from the training duration, the resting heart rate (HR), and the maximal, resting, and mean HRs during the exercise session (10). It presents a multiplicative relationship between the training duration and the HR's variables.

Fatigue can also be considered subjectively (11). Self-reported indicators on judgment scales (such as the Borg rating of perceived exertion scale) are particularly used in endurance sports (e.g., cycling) and appear to be relevant for estimating fatigue during exercise of different intensities (12). However, none of these subjective indicators of fatigue allow us to understand the cognitive process involved in the generation of fatigue. It is therefore essential to develop new approaches that account for the cognitive processes involved in subjective fatigue. We hypothesized that information integration theory [IIT; (13)] can be used to address this shortcoming.

## IIT and the judgment of fatigue

According to IIT, a perception, thought or action depends on the integration of several different types of information. IIT describes the way in which individuals cognitively integrate information to arrive at an overall judgement. Anderson (14) developed the concept of cognitive algebra: the way in which information is combined by an individual will produce additive, multiplicative, and averaging cognitive rules (14). An additive rule is given by a pattern of parallelism. A multiplicative rule, such as a conjunctive rule or a disjunctive rule, is given by a linear fan pattern. When the pattern show a fan open to the left, the cognitive rule is disjunctive, and when the pattern shows a fan open to the left, the cognitive rule is conjunctive. An averaging rule is also given by a parallelism pattern but the concepts of importance weight and prior information are considered (13). In sport, the researchers principally found additive rules and multiplicative rules [for an illustration in sport, see Fruchart (15)].

Graphical methods (based on the response pattern, i.e., the shape of the curves) and statistical methods (mainly repeated-measures analyses of variance) can be used in an IIT analysis. Although IIT has been applied in various domains in sports psychology (e.g., judgments of well-being (15), performance (16), and ethics (17)), its validity in the sports domain is still subject to debate because the experiments do not take place in real-life sporting situations [e.g. (18),]. Indeed, the IIT method is typically based on questionnaires designed by the experimenter and administered in the laboratory. Participants are presented with scenarios based on real sports situations and then have to respond to a question by grading their opinion on a judgment or decision scale.

To the best of our knowledge, only Fruchart et al. (19) have examined the convergent validity of the application of IIT to decision-making patterns for a quick throw-in in handball. The researchers compared the patterns obtained under laboratory conditions (obtained from an IIT-based questionnaire) with the patterns observed in a true sports condition (obtained from a video recording); the two sets of patterns were very similar. This finding demonstrated that the judgment scheme derived in the laboratory study and that inferred from real observations in the sports field were reasonably compatible. However, there are few other studies of this type in the literature. Work on other types of decision or judgment is required to consolidate the validity of an IIT-scenario-based method (13).

IIT scenarios are typically created from the combination of at least two factors that might influence an individuals’ judgment of an indicator (e.g., fatigue). The exercise modalities (e.g., the duration and intensity) are known to determine the onset of fatigue (20, 21). However, the way in which exercise duration and exercise intensity are integrated cognitively during the judgment of fatigue had not previously been investigated.

The primary objective of the present study was to confirm the convergent validity of the IIT method in the judgment of fatigue in sport. To achieve this, we measured the putative associations between information integration data from the laboratory condition, subjective data from the real sports condition, and objective (physiological) data on fatigue from the real sports condition. We also compared the response patterns for cognitive data observed in the laboratory condition with those observed for subjective data in the real sports condition.

Our first hypothesis was that the three types of data (information integration data, subjective data, and physiological data) would be correlated with each other. Our second hypothesis was that exercise duration and exercise intensity would both have a significant effect on the judgment of fatigue. Based on TRIMP, our third hypothesis was individuals would be used a multiplicative integration rule. Lastly, our fourth hypothesis was that the patterns of fatigue as a function of exercise duration and intensity would be similar in the laboratory condition (with information integration data) and the real sports condition (with subjective data).

## Material and method

### Participants

Twenty healthy, experienced cyclists (16 men and 4 women) agreed to take part in the study. The mean number of years of experience (*M_years_*) was 6.3 (*SD* = 4.3). The participants’ age ranged from 19 to 33 (*M_age _*= 23; *SD* = 2.1). The participants did not receive any remuneration for their involvement in the study.

### Material

In the laboratory condition, the participants filled out a questionnaire with six scenarios. Each scenario comprised a cycling situation, a question, and a response scale. The six situations were generated by combining the *duration* factor (two levels: 15 or 30 min) and the *intensity* factor (three levels: 30%, 50% or 70% of the maximal intensity). For each situation, the participants were asked “How tired will you be at the end of this session?”. Each participant then estimated the degree of fatigue at the end of the session on an 11-point rating scale ranging from “Not at all tired” to “Extremely tired” (see Appendix for an example).

In the real sports condition, we transposed these six scenarios into real sports conditions in a sports hall with appropriate equipment (a Wahoo Kickr home trainer). A tablet computer was used for recording the participant's fatigue rating. The participant was equipped with an HR monitor (H10, Polar) and the HR was recorded throughout the exercise session. Each situation's exercise load was calculated by applying Banister's formula (10): TRIMP = T × *Δ*HR × k, where T is the exercise duration (in minutes), ΔHR is (HR_exercise_—HR_rest_)/(HR_max_-HR_rest_), and k is 0.86 $e^{1.67\Delta HR}$ (for a woman) or 0.64 $e^{1.92\Delta HR}$ (for a man).

### Procedure

The study protocol was approved by the independent ethics committee at the University of Toulouse (Toulouse, France; reference: 2022-586). The study included a laboratory condition (which took place in a quiet laboratory room) and a real sports condition (in a sports hall). Each participant was first confronted with the laboratory condition and then with the real sports condition. An initial appointment was arranged with the participant so that he/she could be given information about the study's objectives and procedures and could then give his/her written, informed consent to participation in the study. Each participant kept a copy of the consent form, and another copy was archived by the investigators.

In the laboratory condition, the participant had to read the description of the various cycling scenarios and imagine him/herself performing them. For each scenario, the participant had to judge his/her expected level of fatigue. In line with Anderson's methodology (13), a familiarization phase was followed by an experimental phase. The laboratory session lasted for approximately 10 min and thus provided information on integration data.

At the end of the laboratory session, the investigator made an appointment with the participant so that he/she could take part in the real sports condition. According to the 2 × 3 factorial design described above, each participant performed each of the six real cycling scenarios on separate days and had a three-day interval between sessions. For each real sports situation, the participant performed the physical activity and then indicated his/her level of fatigue on the same scale used in the laboratory condition. The HR was recorded throughout the exercise session. We varied the exercise intensity and duration, using the values from the six laboratory scenarios. The intensity levels were based on the maximal aerobic power (22). The participants were not informed of the exercise duration and intensity values that were to be applied to the session. After the participant had completed the six real sports scenarios, the investigator explained the previously hidden aspects of the protocol. The real sports condition provides us with subjective data and objective (physiological) data.

### Data analysis

The participant’s fatigue rating data from the laboratory condition and the real sports condition were converted into numerical data by calculating the distance between the point on the response scale and the left anchor (the origin).

The numerical data were analyzed statistically and graphically. For each of the three types of data, parametric tests were conducted after the normality of distribution had been checked using the Shapiro-Wilk test. Four repeated-measures analyses of variance (ANOVAs) were conducted according to the *duration*×*intensity* factorial design: the first on information integration data from the laboratory condition, the second on subjective data from the real sports condition, the third on the mean HR data, and the fourth on the TRIMP data. Tukey's *post hoc* tests were performed on the *intensity* factor. We calculated Pearson coefficient for the correlations between the variables. All statistical analyses were performed with Statistica software (version 8).

## Results

### ANOVAs of information integration data from the laboratory condition and subjective data from the real sports condition

**Figure 1:**
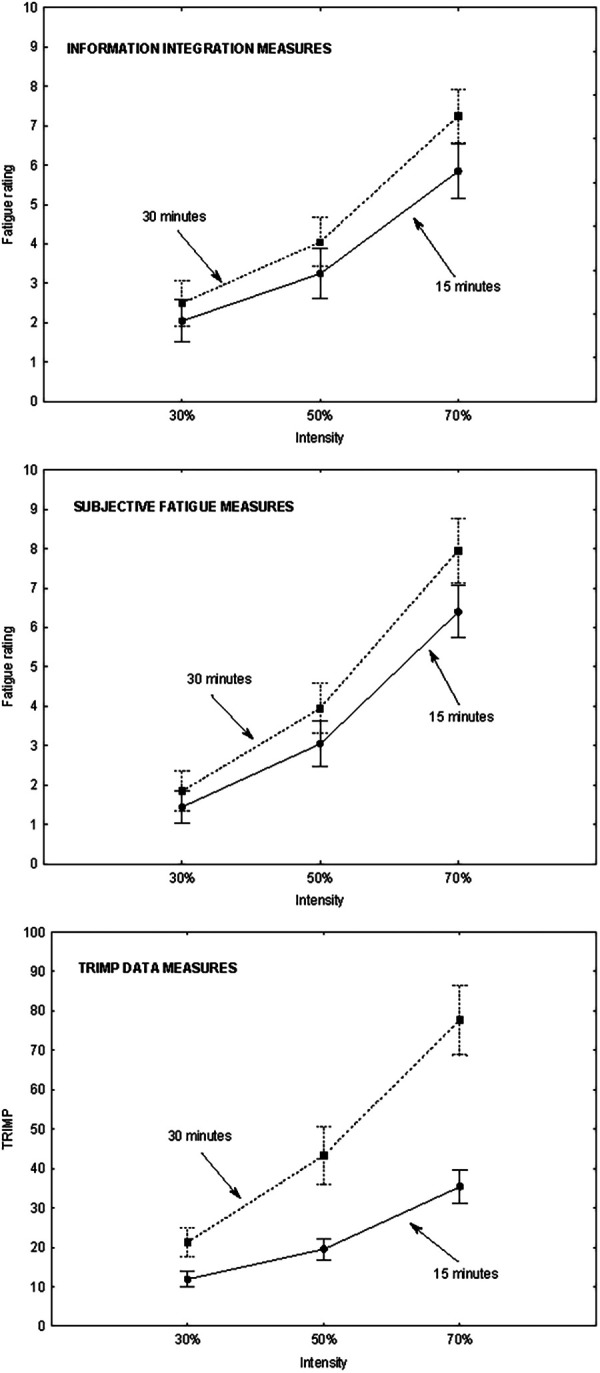
Effect of duration and intensity on the fatigue rating in a laboratory condition (top panel) and in a real sports condition (middle panel) and on the TRIMP in a real sports condition (bottom panel). *Legend*. In the three panels, the three levels of the *intensity* factor are on the x-axis, and each line corresponds to one level of the *duration* factor. In the top and middle panels, the fatigue rating is on the y-axis. In the bottom panel, the TRIMP is on the y-axis.

In a graphical analysis of the data, the curves rise from left to right (indicating an effect of *intensity*) and are separate (indicating an effect of *duration*) (Figure 1). In the laboratory condition (the bottom panel in Figure 1) and in the real sports condition (the middle panel in Figure 1), the curves form a fan opening to the right [indicating that the participants used the same integration conjunctive (multiplicative) rule in both conditions]. The *duration*×*intensity* interaction was statistically significant (*p < *.05) in the laboratory condition [*F*(2, 38) = 3.67, *p = *.035, *η^2^_p_ *= .16] and in the real sports condition [*F*(2, 38) = 7.71, *p = *.002, *η^2^_p_ *= .29] (Tables 1, 2). In both conditions, Tukey's *post hoc* test revealed significant differences (*p* *< *.001) between the three levels of *intensity*.

**Table 1:** Mean and standard deviation values in each scenario for information integration data from the laboratory condition, subjective data, and physiological data (HR mean and TRIMP) from the real motor sports condition.

		Laboratory condition		Real sports condition					
		Information integration data		Subjective data		TRIMP		Mean HR	
Duration	Intensity	*M*	*SD*	*M*	*SD*	*M*	*SD*	*M*	*SD*
15 min	30%	2.05	1.14	1.45	0.89	11.96	4.18	121	12
15 min	50%	3.25	1.33	3.05	1.23	19.58	5.67	139	12
15 min	70%	5.85	1.50	6.04	1.43	35.44	9.29	165	12
30 min	30%	2.5	1.23	1.85	1.09	21.33	7.61	116	13
30 min	50%	4.05	1.32	3.95	1.36	43.34	15.56	143	14
30 min	70%	7.25	1.45	7.95	1.73	77.70	18.95	169	13

**Table 2:** Main results of the ANOVAs of information integration data from the laboratory condition and subjective data and objective (TRIMP) data from the real sports condition.

Factor	Effect		Error				
	*df*	*MS*	*df*	*MS*	*F*	*p*	*η* ^2^_p_
Information integration data from the laboratory condition							
Duration	1	27.08	19	0.81	33.35	<.001	.64
Intensity	2	316.36	38	2.02	156.23	<.001	.89
Duration × intensity	2	3.33	38	0.90	3.67	.035	.16
Subjective data from the real sports condition							
Duration	1	23.41	19	0.32	73.01	<.001	.79
Intensity	2	190.51	38	1.13	168.42	<.001	.90
Duration × intensity	2	2.31	38	0.30	7.71	.002	.29
Objective (TRIMP) data from the real sports condition							
Duration	1	18,946.6	19	93.9	201.85	<.001	.91
Intensity	2	16,297.6	38	59.6	273.38	<.001	.93
Duration × intensity	2	2,718.2	38	49.9	54.50	<.001	.74

The threshold for statistical significance was set to *p* < .05.

### ANOVAs of mean HR data and TRIMP data in the real sports condition

The ANOVA on mean HR data showed that (i) the *duration* factor was not significant, *F*(1, 19) = 1.33, *p = *.30, *η^2^_p_ *= .05; (ii) the *intensity* factor was significant, *F*(2, 38) = 3.67, *p < *.001, *η^2^_p_ *= .96; and (iii) the *duration*×*intensity* interaction was significant, *F*(2, 38) = 3.73, *p = *.03, *η^2^_p_ *= .16 (Table 1). Tukey's *post hoc* test revealed significant differences between the three levels of the *intensity* factor (*p* *< *.001).

With regard to the TRIMP data, the curves rise from left to right (indicates an effect of *intensity*), are separate (indicating an effect of *duration*) and form a fan opening to the right indicating an integration conjunctive (multiplicative) rule (Figure 1, bottom panel). The *duration*×*intensity* interaction was statistically significant (*p < *.05): *F*(2, 38) = 54.50, *p *< .001, *η^2^_p_ *= .74 (Tables 1, 2). Tukey's *post hoc* test revealed significant differences between the three levels of the *intensity* factor (*p* *< *.001).

### Correlations between information integration data from laboratory condition, subjective data from the real sports condition, and objective (physiological) data from the real sports condition

The information integration data from the laboratory condition was correlated with subjective data from the real sports condition and with the objective data (mean HR and TRIMP) from the real sports condition (Table 3). The subjective data from the real sports condition was correlated with the set of objective data (mean HR and TRIMP) from the real sports condition. The mean HR and TRIMP data were correlated with each other.

**Table 3:** Correlations between the information integration data from laboratory condition, the subjective data from the real sports condition, and the objective (mean HR and TRIMP) data from the real sports condition.

	Information integration data from the laboratory condition	Subjective data from the real sports condition	Training impulse (TRIMP)	Mean HR
Information integration data from the laboratory condition	1			
Subjective data from the real sports condition	.729*	1		
TRIMP	.637*	.718*	1	
Mean HR	.619*	.747*	.807*	1

* The threshold for statistical significance was set to *p* < .001.

## Discussion

The primary objective of the present study was to confirm the convergent validity of the IIT method in the judgment of fatigue in sport, using information integration, subjective and physiological data. To this end, we tested the putative association between information integration data from the laboratory condition, subjective data from the real sports condition, and objective (physiological) data from the real sports condition, as a function of the participants’ level of fatigue in different sports situations. We also compared response patterns for the information integration data observed in the laboratory condition and the subjective data observed in the real sports condition.

Our first hypothesis was that the three data sets would be correlated with each other; this was confirmed by the study's results. The physiological data (mean HR and TRIMP) from the real sports condition were correlated with the information integration data from the laboratory condition and the subjective data in the real sports condition. This finding confirms the existence of close relationships between physiological and psychological indicators of fatigue (2). The information integration data from the laboratory condition were also associated with subjective data in the real sports condition. This result supports the correlation found between data observed in a judgment condition (i.e., an information integration condition) and in a real condition (19).

The second hypothesis was that the exercise duration and intensity would have a significant effect on fatigue judgment. In the laboratory condition and in the real sports condition, both exercise duration and intensity were associated with a higher fatigue rating and a higher TRIMP. These findings were in line with literature data on the exercise duration and intensity as indicators of the physiological level of athletes’ fatigue (21). The two factors are also essential in the judgment of fatigue.

Our third hypothesis was that individuals would be used a multiplicative cognitive rule. This was confirmed by the form of patterns. The use of the IIT method and the observed significant intensity-by-duration interactions for all three fatigue measures strongly supports a multiplicative integration rule. Moreover, while the factorial curves in laboratory condition and the factorial curves for subjective estimates of fatigue in real condition are virtually identical, the observed linear-fan pattern (indicating a multiplicative integration rule) is far more pronounced for physiological fatigue. This suggests (i) that participants’ subjective estimates of fatigue mirrored their subsequent (and prior) physical fatigue (as indicated by the significant intercorrelations between these measures), (ii) that exercise intensity and duration are integrated multiplicatively -and physiologically- to determine fatigue (i.e., the relative impact of intensity on fatigue increases with greater duration), and (iii) that this multiplicative integration is particularly pronounced for physical fatigue.

Our fourth hypothesis was that the response pattern of fatigue (as a function of duration and intensity) would be similar in the laboratory condition and the real sports condition (19). Our results confirmed this hypothesis. When judging their level of fatigue, the athletes combined both factors in the same way via a questionnaire presenting a real situation and when performed that activity. This result confirms the validity of IIT found by Fruchart et al. (19) in decision-making for a quick throw-in in handball.

However, our study went further than that reported by Fruchart et al. (19) in the comparison between the response patterns in the laboratory and a real sports condition. Furthermore, we looked at objective (physiological) measures of fatigue. The results based on physiological (TRIMP) data were very similar to those based on information integration data. Exercise intensity and duration both had a significant effect on fatigue. The curves showing the interaction between intensity and duration had the same conjunctive (multiplicative) integration pattern as seen for the information integration data. Further research is required to determine whether physiological integration rules exists in the same way that information integration rules do.

### Limitations and implications

Our study had some limitations, which might open up opportunities for further research. Firstly, it would have been interesting to record other physiological data (e.g., the blood serotonin level) and compare them with the information integration data on fatigue (2). Secondly, we looked at a single type of judgment (i.e., judgment of fatigue); in order to further confirm the validity of IIT in sports, judgment in other situations should be investigated. For instance, one could evaluate whether athletes’ judgments of performance are similar in the laboratory and in a real sports situation. Thirdly, we did not assess the degree of participants’ exercise during each 3-day rest period; such activity could moderate the observed results. Fourthly, we did not study potential moderating effects of athletic fitness and/or skill on this difference between subjective estimates and physiological integration of exercise intensity and duration (for example, would they posit that greater fitness/skill would be expected to reduce this difference, as athletes gain experience with physiological responses to exertion?).

Our results show that (i) when one is careful to use the same factors and rating scales in the laboratory and in the real sports condition and (ii) when the physiological and the rating responses express the same underlying dependent variable (“fatigue”) and the factors in the imaginary setting successfully operationalize the intended determinants of fatigue manipulated in the real setting, it is then possible to directly assess the agreement between the information integration data from the laboratory condition and the physiological and subjective data from the real sports condition.

In terms of IIT, finding overall similar response patterns with ratings and the physiological response is more fundamental than finding a significant correlation between ratings and TRIMP or mean HR. Under the assumption that the ratings provide a linear scale of “fatigue” (meaning an equal-interval scale for the expression of fatigue), finding a similar pattern with TRIMP would signal it as a proper “psychophysiological” (rather than just physiological) index of fatigue, unlike HR. This allows us to contrast the IIT approach to sports fatigue with other rating methods, such as the Borg rating of perceived exertion where there is no “structural criterion” to establish proper “psychophysiological” measure. Of course, the small number of levels used in the design (e.g., only two levels in one of the factors and three in the other) limits the assessment of response linearity in accordance with IIT methodology, which must be merely assumed, rather than established.

Our results may have implications for sports training. Athletes have to monitor a large number of variables before, during and after training (1). The physiological data collected enables coaches to quantify the training load. Although these indicators are highly relevant, they focus solely on the athlete's somatic responses to training. The emergence of judgment indicators would help to (i) take greater account of the cognitive dimension of adaptations to training, (ii) determine the weight given to various factors included in sports situations, and (iii) monitor athletes more closely and provide them with more precise, individualized training programmes. Indeed, IIT studies in the field of sports psychology have shown that an athlete's judgment position (i.e., the manner in which an athlete cognitively combine various pieces of information when judging a situation) depends on his/her characteristics [e.g., (17,23)]. Our results also suggest that while athletes may generally be able to estimate their physical fatigue, they appear to underestimate this fatigue under high levels of both exercise intensity and duration. Coaches should be vigilant about how athletes estimate their level of fatigue. Combining a cognitive-indicator-based IIT method recorded in the laboratory, and physiological and subjective indicators recorded during sports activity might constitute a novel way of monitoring training.

## Data Availability

The raw data supporting the conclusions of this article will be made available by the authors, without undue reservation.

## Appendix

A sample card used under laboratory conditions After a good warm-up, you have been cycling for 30 min at 50% of your maximal aerobic power. How tired will you feel at the end of the session? Not at all tired o—o—o—o—o—o—o—o—o—o—o Extremely tired
